# ^1^H, ^13^C, ^15^N backbone resonance assignment for the 1–164 construct of human XRCC4

**DOI:** 10.1007/s12104-021-10035-6

**Published:** 2021-06-25

**Authors:** Maria Jose Cabello-Lobato, Christine K. Schmidt, Matthew J. Cliff

**Affiliations:** 1 Manchester Cancer Research Centre, Division of Cancer Sciences, School of Medical Sciences, Faculty of Biology, Medicine and Health, University of Manchester, 555 Wilmslow Road, Manchester, M20 4GJ UK; 2 Manchester Institute of Biotechnology (MIB), University of Manchester, 131 Princess St, Manchester, M1 7DN UK

**Keywords:** Backbone resonance assignment, XRCC4, Non-homologous end joining, SUMO

## Abstract

DNA double-strand breaks (DSBs) represent the most cytotoxic DNA lesions, as—if mis- or unrepaired—they can cause cell death or lead to genome instability, which in turn can cause cancer. DSBs are repaired by two major pathways termed homologous recombination and non-homologous end-joining (NHEJ). NHEJ is responsible for repairing the vast majority of DSBs arising in human cells. Defects in NHEJ factors are also associated with microcephaly, primordial dwarfism and immune deficiencies. One of the key proteins important for mediating NHEJ is XRCC4. XRCC4 is a dimer, with the dimer interface mediated by an extended coiled-coil. The N-terminal head domain forms a mixed alpha–beta globular structure. Numerous factors interact with the C-terminus of the coiled-coil domain, which is also associated with significant self-association between XRCC4 dimers. A range of construct lengths of human XRCC4 were expressed and purified, and the 1–164 variant had the best NMR properties, as judged by consistent linewidths, and chemical shift dispersion. In this work we report the ^1^H, ^15^ N and ^13^C backbone resonance assignments of human XRCC4 in the solution form of the 1–164 construct. Assignments were obtained by heteronuclear multidimensional NMR spectroscopy. In total, 156 of 161 assignable residues of XRCC4 were assigned to resonances in the TROSY spectrum, with an additional 11 resonances assigned to His-Tag residues. Prediction of solution secondary structure from a chemical shift analysis using the TALOS + webserver is in good agreement with the published X-ray crystal structures of this protein.

**Supplementary Information:**

The online version contains supplementary material available at 10.1007/s12104-021-10035-6.

## Biological context

Cells are constantly attacked by DNA-damaging agents arising from exogenous (UV light, ionizing radiation, small molecule chemicals in food, drugs and tobacco smoke etc.) and endogenous (e.g. reactive oxygen species arising as metabolic by-products) sources. Consequently, many thousands of DNA lesions are generated in each cell every day (Lieber 2008; Jackson and Bartek 2009). To deal with the damage, cells have evolved complex and intricate pathways collectively termed the DNA damage response that detect the DNA lesions, induce cell cycle checkpoints to allow time for their repair, or induce apoptosis or senescence if the harm is too severe (Jackson and Bartek 2009; Ciccia and Elledge 2010). If left unrepaired, DNA damage can have drastic consequences, as demonstrated for example by certain hereditary DNA repair defects causing cancer predisposition, neurodegenerative disorders, immunodeficiencies and/or premature ageing. DNA double-strand breaks (DSBs) are particularly cytotoxic as their incorrect repair can give rise to chromosomal translocations that can lead to cancer. DSBs are predominantly repaired by two pathways: the first one, termed homologous recombination (HR), repairs DSBs in S/G2 phases of the cell cycle with high fidelity using a homologous sequence, usually the sister chromatid, as a template. The second one, called non-homologous end-joining (NHEJ), can function throughout interphase and is responsible for repairing the majority of DSBs arising in human cells, but with less fidelity compared to HR (Jackson and Bartek 2009; Ciccia and Elledge 2010). XRCC4 is one of around a handful of NHEJ core factors critical for successful end ligations of DSBs (Zhao et al. 2020). NHEJ is initiated by the assembly of the DNA-dependent protein kinase (DNA-PK) holoenzyme on broken DNA ends. DNA-PK comprises DNA-PKcs, the catalytic subunit, and the Ku complex, a heterodimer consisting of Ku70 and Ku80 (Frit et al. 2019). DNA-PK phosphorylates itself and other NHEJ factors such as XRCC4 (Normanno et al. 2017), and acts as a recruitment platform for NHEJ and NHEJ-associated factors. XRCC4 is implicated in promoting NHEJ in several ways. By binding to XLF, XRCC4 is implicated in forming XRCC4-XLF filaments to promote DNA-end synapsis (Jackson and Bartek 2009; Brouwer et al. 2016). Moreover, XRCC4 can bind to the nucleoskeletal protein IFFO1 to promote accurate ligation of broken DNA ends (Li et al. 2019). The most established functions of XRCC4 are to bind to, stabilize, and enhance the activity of LIG4, the downstream DNA ligase required for the final NHEJ step that joins the two broken DNA ends together (Lieber 2008; Jackson and Bartek 2009). While in-depth knowledge is available on the crystal structures of XRCC4 alone and bound to some of its interactors , NMR assignments of this DNA repair protein are lacking, thereby limiting our ability to study lower affinity interactions of XRCC4, not amenable to crystallization, with high resolution.

Human XRCC4 is a 334-amino acid protein (Li et al. 1995) with a globular N-terminal head domain (amino acids 1–115) that folds into a seven-stranded, β-sandwich and a helix–turn–helix motif (Junop et al. 2000). Residues 119–164 comprise a long alpha helical stalk, that forms a homodimeric, parallel, coiled-coil interface with reciprocal interactions from residues 119–155, with a single left-handed crossover. Residues 125–165, and 180–210 have conventional coiled-coil sequences (as assessed by DeepCoil (Ludwiczak et al. 2019)), with residues 165–180 also being helical in crystal structures. Biochemical and crystallographic analysis of full-length isolated XRCC4 points to the existence of a tetrameric aggregation state in equilibrium with a dimeric form of the protein. Sites of protein–protein interactions have been identified at residues 165–180 (Sibanda et al. 2001; Wu et al. 2009) and in the N-terminal domain at residues 103–106 (Ropars et al. 2011). In addition, each helical stalk from residues 119 to 135 is in close contact with strands 1, 2 and 3 of the head domain that belong to the partner subunit, thus rigidifying the connection between the head domain and the stalk in the XRCC4 dimer. The dimer interface is predominantly hydrophobic and is stabilized and kept in register by one salt bridge and two hydrogen bonds. The hydrophobic nature, the extensiveness of the interface and the conservation of residues at the dimer interface from yeast to human all suggest that this crystal dimer persists in solution.

**Fig. 1:**
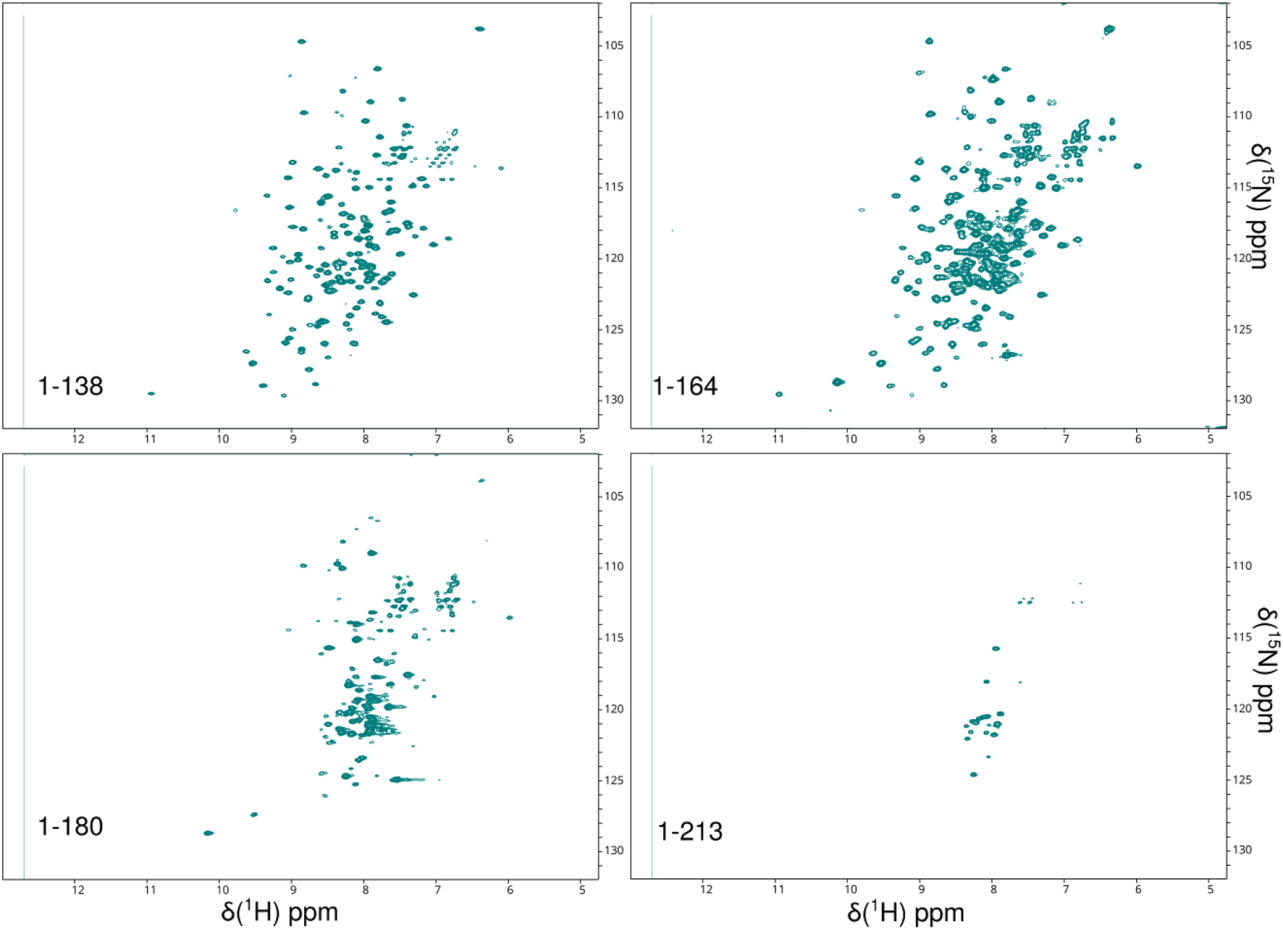
^1^H-^15^ N TROSY spectrum of the XRCC4 1–138, 1–164, 1–180 and 1–213 dimer complex pH 6.8 and 310 K

The heterogeneity of the oligomerisation of the alpha helical stalks is related to their length. In order to obtain well-behaved NMR spectra, a range of constructs was tested, namely full-length XRCC4 (1–334), residues 1–138, 1–164, 1–180 and 1–213. The 1–138 construct represents the minimal length that comprises all the head domain interactions, residues 1–164 comprise the minimal coiled-coil structure observed in crystal structures, and the 164–180 region includes some protein–protein interaction sites. Residues 180–210 complete the regions with strong coiled-coil propensity, and the remaining residues are predicted to be disordered. The optimum solubility and NMR behaviour was found for the 1–164 construct (Fig. 1) and in this work we report ^1^H, ^15^ N and ^13^C backbone resonance assignments of human XRCC4.

## Methods and experiments

### Protein expression and purification

A human XRCC4 gene sequence corresponding to residues 1–164 (with cysteines 93, 128 and 130 mutated to alanines) (Wu et al. 2011) was inserted into a pET-28a plasmid vector linearised with NcoI/BamHI. This plasmid, together with XRCC4 full-length in a pHAT5 plasmid (Wang et al. 2018), XRCC4 residues 1–213 in a pET-15b plasmid (Sibanda et al. 2001) and XRCC4 residues 1–138 in a pET-28a plasmid were generous gifts from Tom Blundell (University of Cambridge, UK). For the triple resonance assignment spectra, the XRCC4 1–164 plasmid was transformed into *Escherichia coli* BL21-CodonPlus (DE3)-RIL (Stratagene) and ^2^H,^15^ N,^13^C-labelled human XRCC4 was expressed in a defined isotopically labelled M9 minimal media containing 99.8% D_2_O, 1 g l^−1 15^ N-ammonium sulphate and 2 g l^−1^ perdeuterated, ^13^C glucose. The cells were grown at 37 °C with shaking until OD600nm = 0.6 and were induced by the addition of 1 mM isopropyl-β-D-thiogalactopyranoside (IPTG). After further incubation for 8 h, cells were harvested by centrifugation at 4000 rpm for 30 min at 4 °C. The cell pellet was resuspended in buffer A (50 mM Tris–HCl pH 8, 300 mM NaCl, 10% glycerol, 10 mM imidazole, 3 mM β-mercaptoethanol) supplemented with cOmplete™ EDTA-free protease inhibitor cocktail (Roche) (one tablet per 50 mL of buffer). The cell suspension was lysed on ice by sonication for 12 cycles of pulsation for 30 s with 30 s cooling intervals. The cell lysate was then separated by ultracentrifugation at 40,000×*g* (17,000 rpm) for 40 min at 4 °C in a Beckman Coulter Avanti JXN-30 centrifuge using a JA 30.50 rotor. Cell lysates were filtered using a 0.22 μM syringe filter and passed through a HisTrap™ FF column (16 × 25 mm, GE HealthCare) equilibrated with 10 column volumes of buffer A. Then, the column was washed with 10 column volumes of buffer A and eluted in buffer A supplemented with 300 mM imidazole. Afterwards, the sample was diluted 6 times in buffer B (20 mM Tris–HCl, 5% glycerol, 10 mM β-mercaptoethanol) and applied to a HiTrap Q HP column (GE HealthCare), washed with 10 column volumes of buffer B supplemented with 50 mM NaCl and eluted in buffer B + 250 mM NaCl. Finally, the XRCC4 solution was concentrated to NMR sample concentrations by a VivaSpin 2 3,000 MWCO centrifugal concentrator and dialysed three times against 1 L of buffer C (20 mM HEPES pH 6.8, 140 mM NaCl, 1 mM EDTA, 2 mM DTT, 100 mM arginine, 100 mM glutamic acid, 0.02% NaN_3_) for 4 h at 4 °C. Protein concentrations were estimated by absorbance at 280 nm (A_280_ = 27,960 M^−1^ cm^−1^). Several backbone amides had not fully exchanged from ^2^ to ^1^H by the end of the preparation procedures. These amides exchanged passively after 15 days at 4 °C, and no further procedure was required to complete exchange, and triple resonance experiments were performed after this period. All reagents including the stable isotopically-labelled compounds ^15^NH_4_Cl (99%), ^13^C_6_,^2^H_7_-D-Glucose (U-13C6, 99%; 1,2,3,4,5,6,6-d7 97–98%) and ^2^H_2_O (99.8%) were purchased with the highest purity from Sigma-Aldrich and used as received. For the initial NMR suitability tests, ^15^ N 1–138, 1–164, 1–180 and 1–213 XRCC4 plasmids were expressed, and the recombinant proteins purified, as described above except for ^12^C-glucose and ^1^H_2_O were used in the defined isotopically labelled M9 minimal media.

### NMR spectroscopy

Protein spectra were recorded at 310 K on a Bruker 800 MHz spectrometer with a ^1^H/^13^C-^15^ N TCI cryoprobe equipped with z-gradients in 20 mM Hepes pH 6.8, 140 mM NaCl, 1 mM EDTA, 2 mM DTT, 100 mM arginine, 100 mM glutamic acid, 0.02% NaN_3_, unless otherwise specified. XRCC4 ^1^H-^15^ N spectra were standard Bruker BEST-TROSY (Favier and Brutscher 2011) with phase-sensitive Echo/Antiecho gradient selection. NMR samples were supplemented with ^2^H_2_O (10% v/v) and trimethylsilyl propanoic acid (TSP; 0.5% v/v) for the deuterium lock and as a chemical shift reference, respectively. Samples were loaded into 5-mm diameter ^2^H_2_O-matched Shigemi NMR tubes. ^1^H chemical shifts were referenced to the internal TSP signal, whereas ^15^ N and ^13^C chemical shifts were referenced indirectly using nuclei-specific gyromagnetic ratios. For the backbone ^1^H, ^15^ N and ^13^C resonance assignment, standard Bruker ^1^H-^15^ N TROSY and TROSY-based 3D versions of HNCA (15%), HNCACB (15%), HN(CO)CACB (8%), HN(CA)CO (18%) and HNCO (9%) experiments (Gardner and Kay 1998) were acquired using non-uniform sampling with a multidimensional Poisson Gap scheduling strategy with sine bell weighting (Hyberts et al. 2013), with the percentage of points collected indicated in parentheses. A 30 Hz (0.15 ppm) resolution in the ^13^C dimension was obtained after processing (zero-filling once). The HNCO spectrum, with one peak per residue in the ^13^C dimension was obtained with 328 hypercomplex points, whereas spectra with two peaks per residue (HNCA, HN(CO)CACB, HN(CA)CO) were obtained with 656 hypercomplex points and the HNCACB spectrum with four peaks per residue was obtained with 1302 hypercomplex points.

### Resonance assignment and data deposition

Backbone ^1^H_N_, ^15^ N, ^13^C_α_, ^13^C_β_ and ^13^C’ resonances were assigned for human XRCC4 dimer using standard triple resonance methodology (Gardner and Kay 1998). Spectra were processed with TopSpin software version 3.5. Peak picking and frequency matching was performed within CCPNMR Analysis version 2.5 (Vranken et al. 2005). Additional confidence in the assignment was gained by comparison between the ^1^H-^15^ N TROSY spectra of different constructs, with the shorter 1–138 version (see Fig. 1) having equivalent peaks for 136/138 resonances. The backbone ^1^H_N_, ^15^ N and ^13^C chemical shifts have been deposited in the BioMagResBank (http://www.bmrb.wisc.edu/) under the BMRB accession code 50,742. The human XRCC4 construct used in this study including the N-terminal His-Tag results in the XRCC4 sequence starting with M1-G2-S3-S4-…, but the residue numbering is defined as M1-E2-R3-K4…, so the N-terminal His-Tag is given negative numbers starting -31, which has been used here throughout.

**Fig. 2:**
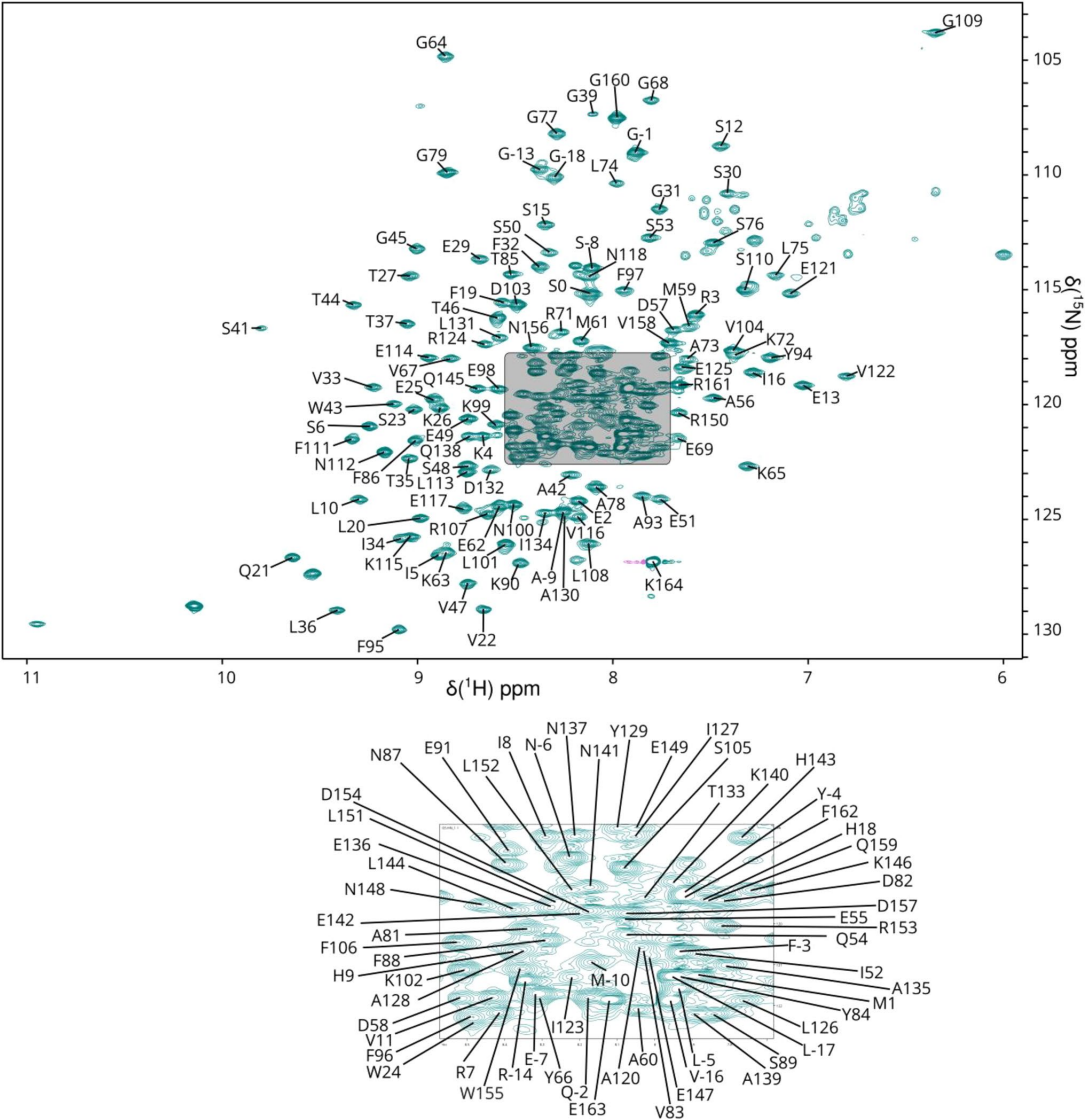
^1^H-^15^ N TROSY spectrum of the XRCC4 1–164 dimer complex at pH 6.8 and 310 K. The assignments of backbone amide resonances are indicated by residue type and sequence number. The lower panel shows the detail of the shaded square in the upper panel

**Fig. 3:**
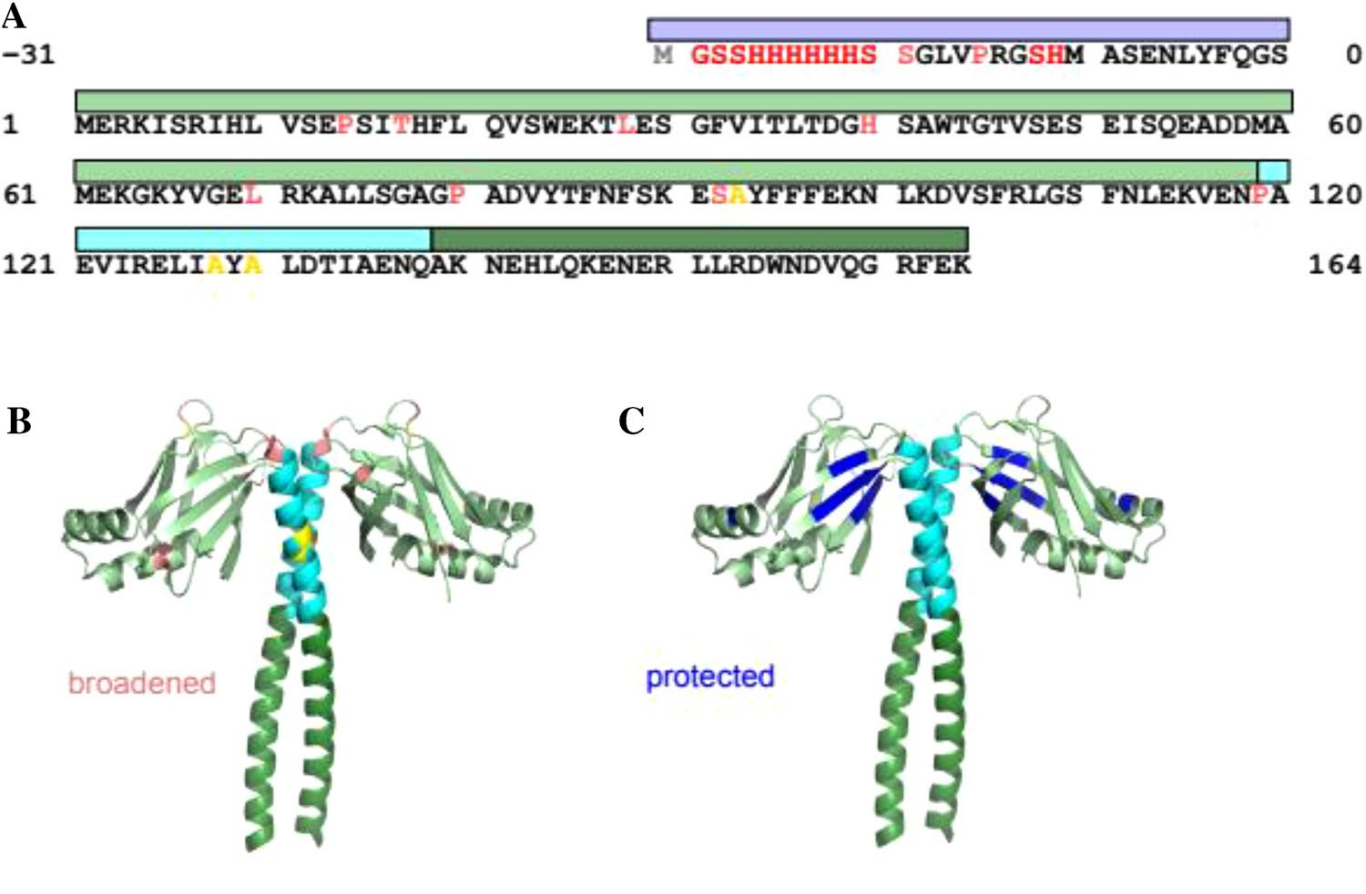
Sequence (**a**) and structural (**b**) context of assignment completeness, and structural context of amide hydrogen protection (**c**). **a** Coloured bars represent HisTag (blue), N-terminal domain (light green), initial helical region (cyan) and conventional coiled-coil domain (dark green). Sequence is coloured red for unassigned, salmon for part assigned (no HN), black for fully assigned residues. Additionally, positions of cys-ala mutations are coloured yellow (all 100% assigned). **b** is a cartoon representation of the crystal structure 1ik9, truncated at residue 164, and follows the same colouring as (**a**). **c** is the same cartoon representation of the crystal structure (pdb entry 1ik9, Sibanda et al. 2001), but with the hydrogen exchange protected amides coloured dark blue

Excluding the 3 proline residues and the N-terminal methionine from the 195-residue XRCC4 1–164 protein sequence, 167 out of a total of 192 residues were assigned in the ^1^H-^15^ N TROSY spectrum of the XRCC4 dimer (Fig. 2). 20 of the unassigned residues are in the His-Tag. Excluding the His-Tag, 96% of all backbone resonances were assigned (97% of ^1^H_N_, 95% of ^15^ N, 100% of ^13^C_α,_ 99% of ^13^C_β_ and 88% of ^13^C’ nuclei). There are 5 residues that remain unassigned in the ^1^H-^15^ N TROSY spectrum (T17, L28, H40, L70 and S92, Fig. 2) and their ^1^H-^15^ N TROSY correlations are likely to be attenuated beyond detection by either fast exchange with solvent or intermediate exchange broadening on the millisecond timescale (Fig. 3). L28 and L70 colocate in the crystal structures, suggesting that intermediate exchange is responsible for the broadening in this area. The amides of some residues were resistant to hydrogen–deuterium exchange, and remained as ^2^H in protonated buffer for over a week after being prepared in deuterated media. These residues were H18-Q22, V33-T37, W43, T44 and E55. The residues are all in one β-sheet (formed by strands 2, 3 and 4), except for E55 is in the core of helix 2 (Fig. 3). The amides exchanged passively after 15 days at 4 °C.

**Fig. 4:**
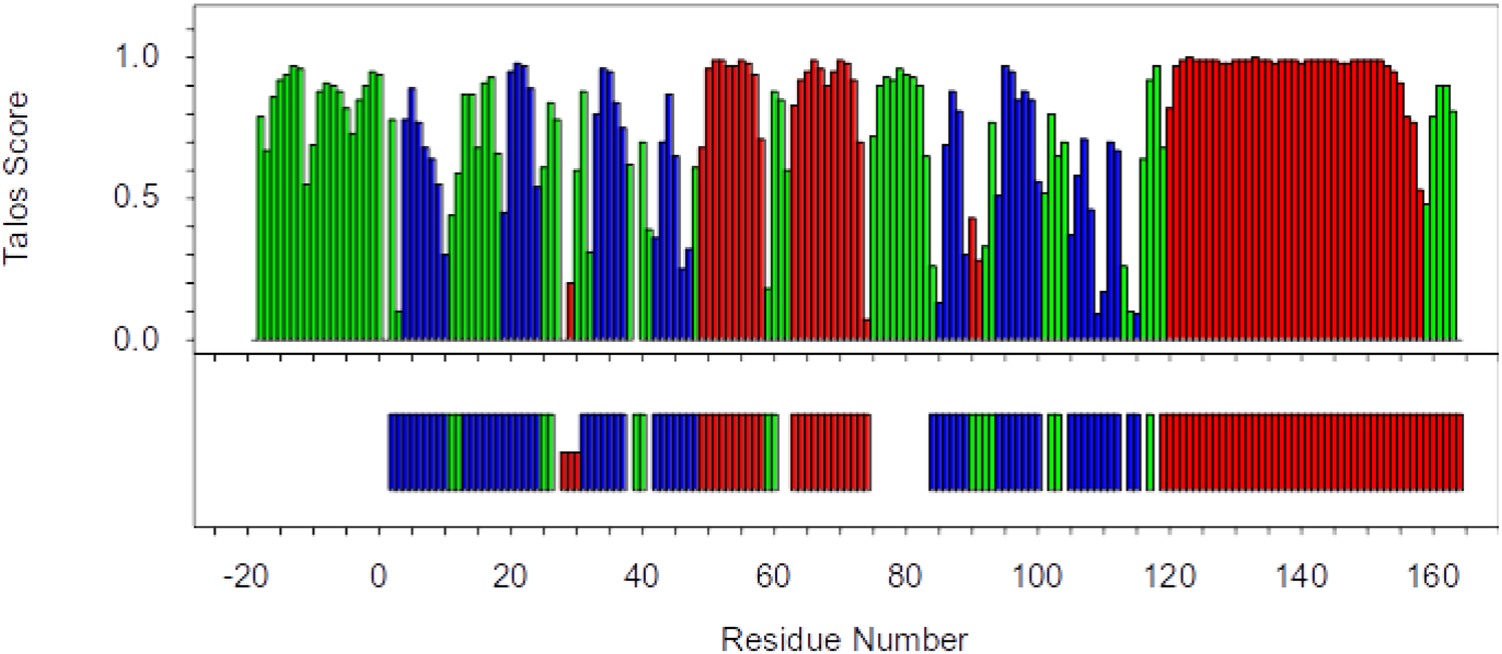
TALOS + . Comparison of TALOS + secondary structure prediction (and score, top) with the crystal structure (PDB code 1ik9; bottom), coloured green for loops, blue for strand and red for helix. Crystal structure secondary structure assignment by DSSP (Frishman and Argos 1995). Residue numbering as in Fig. 2a and explained in text

The secondary structure content of XRCC4 was predicted by uploading the backbone ^1^HN, ^15^ N, ^13^C_α_, ^13^C_β_ and ^13^C’ chemical shifts of the XRCC4 1–164 dimer construct to the TALOS + webserver (Shen et al. 2009). Figure 4 illustrates the comparison between the predicted secondary structure for the solution structure and the secondary structure present in the crystal. These data are in very good agreement, which indicates that the solution conformation is similar to the protein structure observed in the crystal, and provides confidence in the assignments of the XRCC4 dimer.

## Supplementary Information

Below is the link to the electronic supplementary material.

Supplementary file1 (SVG 1486 kb)

Supplementary file2 (SVG 1062 kb)

Supplementary file3 (SVG 458 kb)

Supplementary file4 (SVG 207 kb)

## Data Availability

NMR assignments are deposited in the BMRB with accession code 50742.

## Code availability

No new code arises from this work.
